# Salivary Alpha Amylase as a Noninvasive Biomarker for Dental Fear and Its Correlation with Behavior of Children during Dental Treatment

**DOI:** 10.5005/jp-journals-10005-1227

**Published:** 2014-04-26

**Authors:** Hina Noorani, Hrishikesh V Joshi, PK Shivaprakash

**Affiliations:** Professor and Head, Department of Pediatric and Preventive Dentistry, PM Nadagouda Memorial Dental College and Hospital, Bagalkot Karnataka, India; Postgraduate Student, Department of Pediatric and Preventive Dentistry, PM Nadagouda Memorial Dental College and Hospital, Bagalkot Karnataka, India; Professor, Department of Pediatric and Preventive Dentistry, PM Nadagouda Memorial Dental College and Hospital, Bagalkot Karnataka, India

**Keywords:** Salivary alpha amylase, Child fear survey schedule-dental subscale, Dental fear

## Abstract

**Objective: **Objectives of our studies were to predict dental fear in a child patient depending on salivary alpha amylase (sAA) level before and after dental treatment and to evaluate correla­tion of later with behavior of child patient during dental treatment.

**Materials and methods: **Seventy-seven children between age of 5 and 12 years were divided in three groups. Group 1 consisted of 25 school children who did not undergo any dental treatment. Groups 2 and 3 underwent dental treatment without and with local anesthesia respectively. Groups 2 and 3 were administered child fear survey schedule-dental subscale (CFSS-DS) questionnaire before treatment. Salivary samples were collected for sAA estimation in groups 2 and 3 children before and after completion of dental treatment and behavior during treatment was noted using Frankel behavior rating scale. Group 1 acted as control in which salivary sample was collected in absence of dental stress.

**Results: **When groups 2 and 3 were combined, pretreatment sAA level had a statistically significant (p = 0.0094) correlation with CFSS-DS scores.

**Conclusion: **Alpha amylase can be used as a screening tool to predict level of dental fear in a child patient.

**How to cite this article: **Noorani H, Joshi HV, Shivaprakash PK. Salivary Alpha Amylase as a Noninvasive Biomarker for Dental Fear and Its Correlation with Behavior of Children during Dental Treatment. Int J Clin Pediatr Dent 2014;7(1):19-23.

## INTRODUCTION

The dental fear, an issue in dentistry is magnified by the high prevalence of dental fear reported in many countries. Literature has reported variable prevalence of dental fear (3-43%) in children from various countries which is a major cause of delay or avoidance of dental treatment in child population.^1^ So identification of such children before starting dental treatment may be helpful for a pedodontist to plan the treatment strategy more Efficiently and develop rapport with the patient.

From many years research is being carried out on use of salivary biomarkers to predict dental anxiety and fear. Most commonly investigated salivary biomarkers for relationship with dental fear are salivary cortisol and chromogranin A. Some studies have reported positive correlation of salivary cortisol with dental fear^2^ but some studies fail to do so.^3^Research is being done on salivary chromogranin A and dental fear but no correlation has yet been established.^4^ So, a continuous effort is being made by Scientific community to find a reliable biomarker for dental fear.

Recent years have shown an increasing interest in salivary alpha amylase (sAA) as a noninvasive marker for sympathetic nervous system activity which reflects fear in a person. Studies have shown that sAA activity is increased during stress.^5^ Even extensive studies have been done in field of psychoneuroendocrinology to evaluate importance of sAA activity and currently sAA is considered a reliable marker of sympathetic nervous system activity as a result of stress and fear^5,6^ So, there is a possibility that even dental fear should cause significant changes in sAA level as it reflects sympathetic nervous system activity. Our study is an effort to investigate changes in sAA levels related to dental fear in a child dental patient and its possible role in predicting behavior during dental treatment.

## MATERIALS AND METHODS

A total of 77 children were studied after dividing them in three groups. Group 1 consisted of 25 healthy children between ages of 5 and 12 years who were selected from a school who acted as a control group. Groups 2 and 3 children were selected from the outpatient department. Group 2 con­sisted of 25 children who needed dental treatment without use of local anesthetic injection while group 3 consisted of 27 children whose treatment needed administration of local anesthetic injection.

**Fig. 1:**
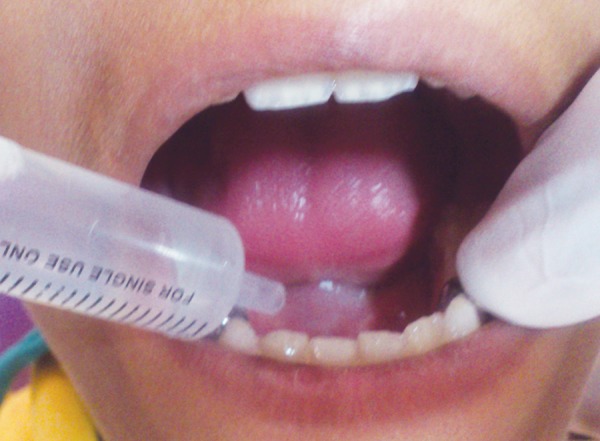
Oral cavity rinsed with deionized water

**Fig. 2:**
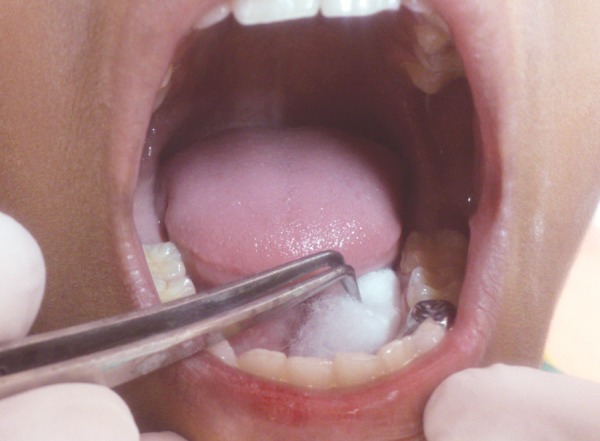
Cotton pledget placed below the tongue

**Fig. 3:**
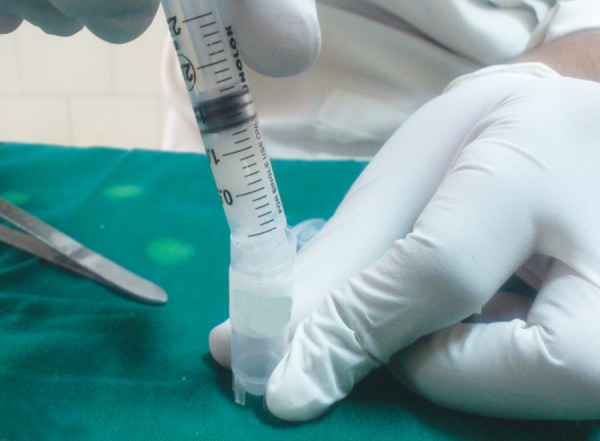
Cotton pledget placed in syringe and saliva expressed in sterile container

A detailed medical history was taken to rule out any systemic disorder in all the children. Saliva was collected from all children between 10.00 am and 12.00 pm to avoid circadian variation. Children of group 1 were uninformed about the study before saliva sample collection as it may have induced fear in them which can affect sAA levels. In these children, first oral cavity was thoroughly washed with deionized water to wash away previously collected saliva in oral cavity (Fig. 1). Fresh unstimulated whole saliva was collected by method described by Harmon et al^7^ in which a cotton pledget is placed below the tongue (Fig. 2). When cotton pledget got fully saturated with saliva, it was removed and placed in an empty syringe with piston removed. By pressing piston, approximately 1 ml of saliva was expressed from cotton and collected in sterilized container (Fig. 3). It was stored at –20°C till it is sent to the laboratory for analysis of alpha amylase.

Children of groups 2 and 3 were instructed not to eat or drink anything 1 hour before procedure^8^ on the day of saliva sample collection. Before starting treatment, every child was given child fear survey schedule dental subscale (CFSS-DS) questionnaire.^9^ Assistance was given to younger children either by parents or by operator in understanding and marking the various components of scale. After completion of the questionnaire saliva sample was collected from child using same procedure described by Harmon et al.^7^

After collecting saliva sample required dental treatment was performed for the child. After the completion of dental treatment another saliva sample was collected by using same method described above. Then behavior of child during dental treatment was assessed using dentists question­naire having Frankl behavior rating scale by the operator. To avoid bias in study, all children were treated by same operator so behavior management strategies used remained fairly constant.

Then the sAA level in saliva before and after dental treatment was compared with dental fear and behavior of the child during dental treatment using various statistical tests.

## RESULTS

Mean CFSS-DS score for total sample was 28.6730 with standard deviation (SD) of 10.4327. Males had mean CFSS-DS score of 30.5769 (SD = 10.8524) while females had mean CFSS-DS score of 26.7692 (SD = 10.2681). When males and females were compared using t-test with respect to CFSS-DS scores the difference was statistically insignificant with p-value of 0.1996 and t-value 1.2999.

Mean pretreatment sAA level in group 1 was 109.60 IU; in group 2 it was 120.81 IU while in children of group 3 pretreatment sAA was 133.10 IU. When sAA levels in three groups was compared with each other using one way ANOVA test the difference was found to be statistically insignificant with p-value of 0.7533.

When groups 2 and 3 were combined, pretreatment sAA level had a statistically significant (p = 0.0094) correlation with CFSS-DS scores when compared using Karl Pearson's coefficient method. Similarly, even post-treatment sAA levels had a high statistically significant correlation (p = 0.0000) with CFSS-DS scores (Table 1).

In group 3, pretreatment sAA levels had statistically significant relationship with CFSS-DS scores with a p-value of 0.0001 while post-treatment sAA also had p-value of 0.0000 proving highly statistically significant relation with CFSS-DS score. In group 2, pretreatment sAA did not have statistically significant correlation (p = 0.5890) with CFSS-DS scores when compared by Karl Pearson's coefficient method while post-treatment sAA had statistically significant correlation (p = 0.0005) with CFSS-DS scores (*see *Table 1).

Mean pretreatment sAA level in group 2 was 120.8120 IU (SD = 100.83) and in group 3, it was 133.100 (SD = 159.84). When these two were compared using t-test no statistically significant difference was observed with p-value of 0.7396. Mean post-treatment sAA level in group 2 was found to be 204.8860 IU (SD = 209.79); while in group 3, it was 267.9778 IU (SD = 282.56). A p-value of 0.3681 was obtained after comparing these two by using t-test which is statistically insignificant difference. The difference between pre- and post-treatment sAA levels in groups 2 and 3 was also analyzed using student t-test which gave statistically insignificant results with a p-value of 0.3003.

**Graph 1:**
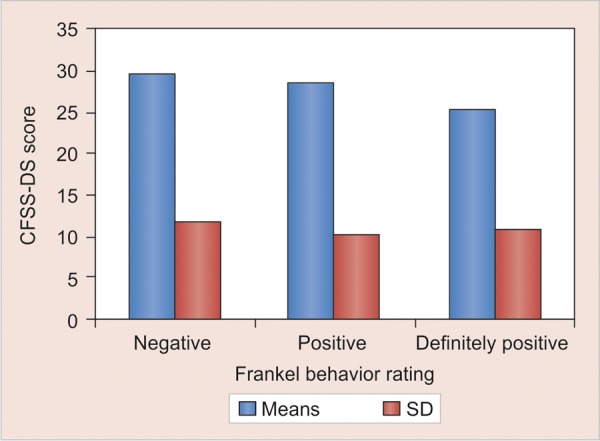
Comparison of CFSS-DS scores with behavior of children

Mean CFSS-DS score in children showing negative behavior was 29.625 (SD = 11.713). In children with positive behavior mean CFSS-DS score was 28.594 (SD = 10.323) while mean CFSS-DS score in children with definitely positive group was 25.500 (SD = 10.786).When behavior of children was compared with CFSS-DS scores of children using ANOVA test statistically insignificant results were obtained with p-value of 0.79079 (Graph 1).

## DISCUSSION

Generally, it is considered that sympathetic stimulation [via norepinephrine (NE)] leads to high levels of protein concentrations, e.g. alpha amylase, whereas high rates of fluid output occur in response to parasympathetic cholinergic stimulation [via acetylcholine (ACh)]. But one recent study^10^has proved that with regard to the secretion of alpha-amylase, the two branches of the autonomic nervous system do not act independently as both parasympathetic and sympathetic activation lead to an increase in alpha-amylase levels. So, the major determinant of sAA secretion will be the total activity of autonomic nervous system.

The emotional stimulation of fear is also discharged by the autonomous nervous system through hypothalamus. However, in humans this activation of ANS in turn leading to hypothalamus stimulation can be modified by cortical interferences so that a man with his highly developed cortex can control his emotions of fear, to a degree, through rationalization and determination. A young child, with less developed cortex is unable to rationalize much and therefore develops fear easily as compared to an older child or adult who has more cortical development. So, as the mental age of child increases these responses of fear can be controlled more and more by cortex through higher psychic functions. Hence, depending on degree of rationalization of child degree of fear response will change, which in turn will change degree of ANS activation. And, as ANS controls secretion of alpha amylase, ultimately it affects sAA levels. So, according to degree of fear present in a child, even the sAA secretion should also change.

Our study also showed similar relation except in children of group 3. The statistically insignificant relationship of CFSS-DS scores with pretreatment levels in group 3 may be due to the fear of injection present in many children. A study by Milgrom P et al^11^ showed that in about 25% of adults fear of injection is present. Another study^12^ in children showed presence of needle phobia in 19% of children between 4 and 6 years of age which decreased to 11% in 10 to 11-year-old children. Though needle phobia is present in only 11 to 20% of children, fear of injections which is less severe form of phobia may affect many children in this study sample. As above-mentioned data gives prevalence of fear of injection in adults as 25%, values might be higher in children if we consider degree of emotional development in them. As children were informed about treatment to be given before administration of CFSS-DS questionnaire, prospect of receiving injection may have heightened the fear response in a child and disrupted the relationship between alpha amylase and fear specifically in this group.

**Table 1:** **Table 1: **Correlation between CFSS-DS scores with pre- and post- treatment sAA by Karl Pearson's correlation coefficient method

*Samples*		*Time point*		*Correlation between fear (CFSS) scores with*			
				*Correlation* *coefficient*		*p-value*	
Total (combined)		Pretreatment sAA		0.3567		0.0094*			
		Post-treatment sAA		0.7472		0.0000*			
Group 2		Pretreatment sAA		0.1088		0.5890			
		Post-treatment sAA		0.6235		0.0005*			
Group 3		Pretreatment sAA		0.6872		0.0001*			
		Post-treatment sAA		0.9673		0.0000*			

* Statistically significant correlation p < 0.05

In our study, control group was mainly designed to obtain value of sAA when the child is free from any mental and physical stress, i.e. in relaxed state with no dental treatment to be carried so that we can compare it with sAA values under dental fear and treatment stress. So children in this group were not informed about the study prior to sample collection as well as on day and time of saliva collection. It was care­fully decided so that no exams or physical education class was coinciding with the time of saliva collection. Though these precautions were taken, stress cannot be absolutely eliminated. The threshold of stress or mental excitation may vary from child to child depending on his mental and physical development. So, a simple act of reading a poem, solving a mathematical problem, small fight with a peer or constant teasing by others can act as a stress and can increase sAA values in control group. Even in study groups, only medical history was taken to rule out other factors causing change in sAA levels. Even in these children other factors like stress of exam, fights between parents, constant family problems or minor illness like common cold can also act as a stress and can increase pretreatment sAA values in some children. So, an absolute stress free or relaxed state cannot be obtained in clinical settings which is a major limitation of this study and may have affected values of pretreatment alpha amylase and those in control group.

The pre- and post-treatment alpha amylase values were compared between males and females but the difference was statistically insignificant. This result is in accordance with previous studies^8^ which do not show any difference in sAA levels related to sex. Even no different Profile of alpha amylase secretion has been proved to be present in males and females to acute stress response till now. The only variation seen in sAA levels related to sex is during pregnancy which attenuates stress response for secretion of alpha amylase.

Behavior of children during dental treatment was also compared in this study with other variables. When different behaviors were compared with pre- and post-treatment sAA in combined sample from groups 2 and 3, it was found that in post-treatment alpha amylase levels a statisti­cally significant relationship was found between various behavior groups. But, this comparison showed statistically significant increase in alpha amylase level as behavior of child improved, i.e. children showing positive behavior had more alpha amylase values as compared to children showing negative behavior. This inverse relationship may be due to complex interplay of factors which affect behavior of child during dental treatment. Apart from dental fear other factors affecting behavior of child are parental attitude, environment of dental clinic, reception of child by dental personnel, rapport with dentist past medical/ dental experiences and growth and development of child.^13^ So, these factors may vary from child to child which can result in this inverse relationship.

One other possible explanation for this relationship is that children who show positive behavior may require more mental activity to overcome their fears and cooperate with the dentist rather than just expressing the fears and show a negative behavior in dental operatory. Children showing negative behavior show less mental activity as they are just expressing their primal emotion of fear without any attempt of brain cortex to control this emotion which resulted in lower sAA values.^14^ So, overcoming fear may require more mental activity by cortex of brain which may subsequently cause more stimulation of ANS which will result in higher alpha amylase levels in children with more positive behavior.

Similar to above results, even a statistically significant correlation was observed between various behaviors of children in group 3 and post-treatment alpha amylase levels which can also be explained with above described factors. But when same was compared in group 2 which involved treatment without administration of local anesthetic injection statistically insignificant correlation was obtained. But, if we compare pretreatment sAA values with behavior a definite decrease in alpha amylase can be observed with improve­ment in child behavior. This can be due to the less severe nature of dental treatment involved in this group which was a lesser stress to child as compared to treatments in group 3 which were of more invasive nature. So, it can be suspected that among previously explained factors severity of stress, i.e. here type of treatment may significantly affect the level of sAA compared to other factors like maternal attitude, previous dental experiences, etc.

In studies, it is proved that behavior of a child in dental operatory improves as dental fear in him decreases.^9^ It means that in children as CFSS-DS score drops behavior should change to a more positive behavior as compared to children with higher CFSS-DS values. Even similar pattern was seen in our study. Though this difference was not statistically significant, it can be suspected that other factors affecting behavior and dental fear might have acted as confounding factors and affected results.

So, within the limitations of our study, we conclude that sAA can be a valuable screening tool for dental fear in children and it can be a valuable aid in diagnosing and treating dental fear in a child.
